# Supplementary material for: Development of Exhaustion and Acquisition of Regulatory Function by Infiltrating CD8+CD28− T Lymphocytes Dictate Clinical Outcome in Head and Neck Cancer
Source: Cancers (Basel). 2021 May 6;13(9):2234. doi: 10.3390/cancers13092234 (PMC8124419; doi:10.3390/cancers13092234)
Supplement: Supplementary file 1 [file cancers-13-02234-s001.zip › cancers-1139513-supplementary.pdf]

cancers

MDPI

Article

Supplementary Materials: Development of Exhaustion and Acquisition of Regulatory Function by Infiltrating CD8+CD28- T Lymphocytes Dictate Clinical Outcome in Head and Neck Cancer

Daniela Fenoglio, Liliana Belgioia, Alessia Parodi, Francesco Missale, Almalina Bacigalupo, Alison Tarke, Fabiola Incandela, Simone Negrini, Stefania Vecchio, Tiziana Altosole, Sara Vlah, Giuseppina Astone, Francesca Costabile, Alessandro Ascoli, Francesca Ferrera, Guido Schenone, Raffaele De Palma, Alessio Signori, Giorgio Peretti, Renzo Corvò, and Gilberto Filaci

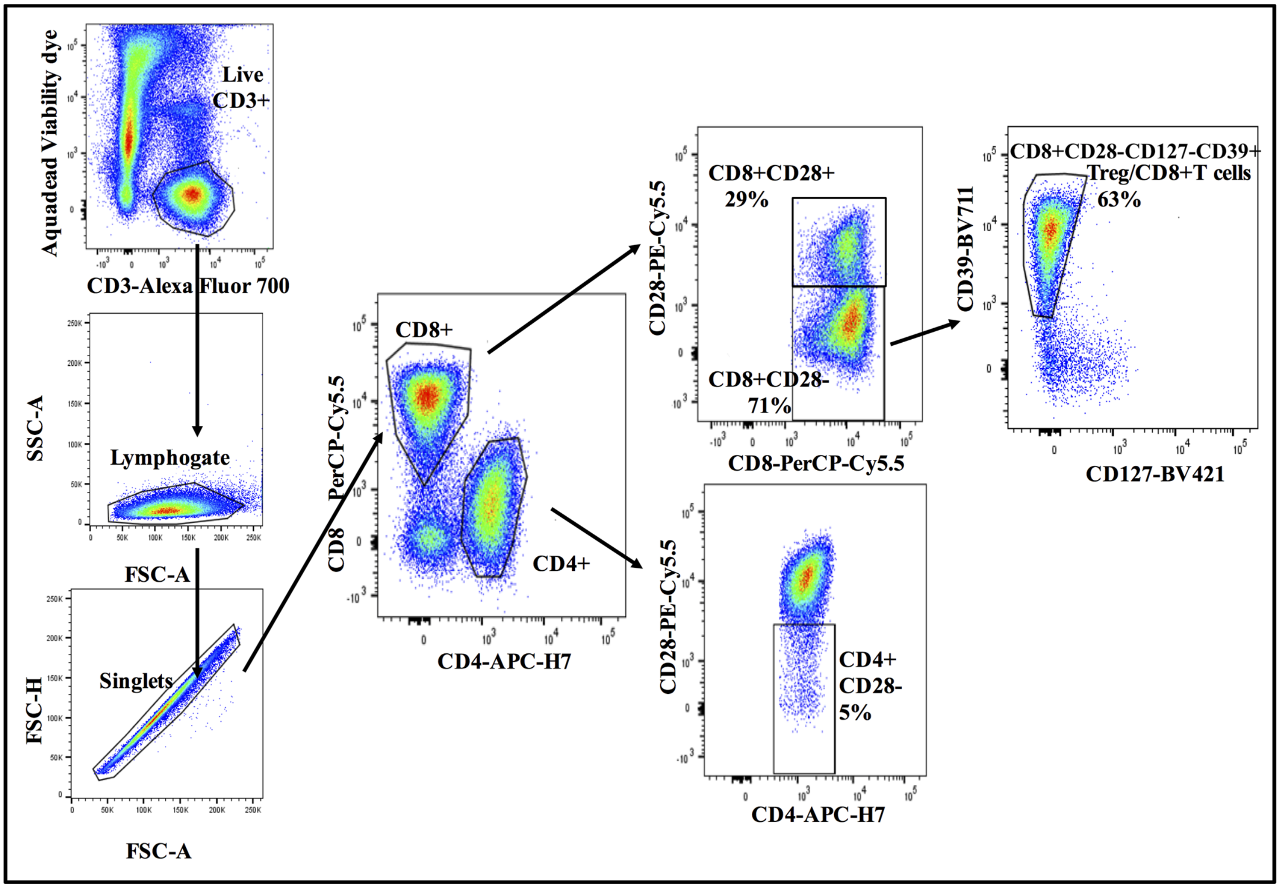

Figure S1. Phenotypic characterization of intratumoral CD8+CD28-CD127-CD39+ Treg. Strategy of gating for identifying CD8+CD28-$CD127^{l}$-CD39+ Treg. Data refer to the representative analysis performed with cells from the tumor specimen of patient #28.

Table S1. T cell subpopulations analyzed in the study.

Type of Marker	Total T Lymphocyes	CD4+ T Lymphocytes	CD8+ T Lymphocytes
Lineage	CD3+	CD3 + CD4+	CD3 + CD8+
Maturation stage		CD4+CD45RA+CCR7+ (naïve)	CD8+CD28+<br>CD8+CD28-

	CD4+CD45RA-CCR7+ (CM)<br>CD4+CD45RA-CCR7- (EM)	CD8+CD28+CD45RA+CCR7+ (naïve)<br>CD8+CD28+CD45RA-CCR7+ (CM)
	CD4+CD45RA+CCR7- (TEM)	CD8+CD28-CD45RA-CCR7- (EM)
		CD8+CD28-CD45RA+CCR7- (TEM)
Regulatory function	CD4+CD25^{hi}FoxP3+ (CD4+ Treg)	CD8+CD28-CD127-CD39+ (CD8+ Treg)
	CD4+CD25^{hi}FoxP3+PD-1+	CD8+CD28-CD127-CD39+ PD-1+
	CD4+CD25^{hi}FoxP3+CD152+	
	CD4+CD25^{hi}FoxP3+CD39+	
Inhibitory receptor	CD4 + PD-1+	CD8+PD-1+
	CD4+CD39+	CD8+PD-1-
	CD4+ CD39+ PD-1+	CD8+CD152+
	CD4+ CD152+ PD-1+	CD8+CD39+
		CD8+PD-1+CD152+
		CD8+PD-1+CD39+
